# HEART Score Distribution and Association with In-Hospital Management and Outcomes in COVID-19 Survivors Presenting with Acute Chest Pain

**DOI:** 10.3390/jcm15187088

**Published:** 2026-09-13

**Authors:** Tiberiu Buleu, Mircea Iurciuc, Ovidiu Alexandru Mederle, Daian-Ionel Popa, Ion Petre, Gheorghe Nicusor Pop, Codrina Mihaela Levai, Răzvan Roșca, Ana Maria Pah, Carmen Gabriela Williams, Maria Laura Craciun, Dana Emilia Movila, Catalin Prodan-Barbulescu, Aida Iancu, Florina Buleu

**Affiliations:** 1 Faculty of General Medicine, “Victor Babes” University of Medicine and Pharmacy, 300041 Timisoara, Romania; tiberiu.buleu@umft.ro; 2 Department of Clinic Nursing, “Victor Babes” University of Medicine and Pharmacy, Eftimie Murgu Square 2, 300041 Timisoara, Romania; iurciuc.mircea@umft.ro; 3 Department of Surgery I, Emergency Medicine Discipline, “Victor Babes” University of Medicine and Pharmacy, 300041 Timisoara, Romania; mederle.ovidiu@umft.ro; 4 Emergency Department, Emergency Municipal Clinical Hospital, 300254 Timisoara, Romania; williams.carmen@umft.ro; 5 Research Center for Medical Communication, Victor Babes University of Medicine and Pharmacy, 300041 Timisoara, Romania; codrinalevai@umft.ro; 6 Department of Functional Sciences, Victor Babes University of Medicine and Pharmacy, E. Murgu Square No. 2, 300041 Timisoara, Romania; 7 Center for Modeling Biological Systems and Data Analysis (CMSBAD), “Victor Babes” University of Medicine and Pharmacy, 300041 Timisoara, Romania; pop.nicusor@umft.ro; 8 Department for the Prevention and Control of Healthcare-Associated Infections (CPIAAM), Clinical Hospital of Infectious Diseases and Pulmonology “Dr. Victor Babeș” Timișoara, Gheorghe Adam Street No. 13, 300310 Timișoara, Romania; razvan.rosca@rezident.umft.ro; 9 Department of Cardiology, “Victor Babes” University of Medicine and Pharmacy, 300041 Timisoara, Romania; anamaria.pah@umft.ro (A.M.P.); laura.craciun@umft.ro (M.L.C.); man.dana@umft.ro (D.E.M.); florina.buleu@umft.ro (F.B.); 10 Research Centre of Timisoara Institute of Cardiovascular Diseases, “Victor Babes” University of Medicine and Pharmacy, 300041 Timisoara, Romania; 11 Department I, Discipline of Anatomy and Embriology, “Victor Babes” University of Medicine and Pharmacy, 300041 Timisoara, Romania; catalin.prodan-barbulescu@umft.ro; 12 2nd Surgery Clinic, Timisoara Emergency County Hospital, 300723 Timisoara, Romania; 13 Doctoral School, “Victor Babes” University of Medicine and Pharmacy Timisoara, Eftimie Murgu Square 2, 300041 Timisoara, Romania; 14 Department of Radiology, Victor Babes University of Medicine and Pharmacy, E. Murgu Square No. 2, 300041 Timisoara, Romania; aida.parvu@umft.ro

**Keywords:** COVID-19 survivors, HEART score, acute chest pain, emergency department, acute coronary syndrome, cardiac troponin, risk stratification, coronary revascularization, cardiovascular risk

## Abstract

**Background:** Acute chest pain is a common cause for presentation to the emergency department (ED), where rapid identification of patients at high risk for acute coronary syndrome is essential. The HEART score combines medical history, electrocardiogram, age, risk factors, and troponin levels to provide structured risk stratification. However, evidence regarding the HEART score in COVID-19 survivors presenting with acute chest pain remains limited. This study examined the distribution of HEART scores and their associations with decisions made in the emergency department, hospital management, rises in cardiac troponin levels, coronary revascularization, and in-hospital mortality. **Materials and Methods:** In this single-center retrospective observational study, 400 consecutive adult COVID-19 survivors who presented to the ED with acute non-traumatic chest pain were included. HEART scores were retrospectively calculated from routinely available clinical and laboratory data, and patients were categorized as low (0–3), moderate (4–6), or high risk (7–10). **Results:** The median HEART score was 2 (IQR 0–5); 264 patients (66.0%) were classified as low risk, 50 (12.5%) as moderate risk and 86 (21.5%) as high risk. The distribution of the total score was bimodal, with 128 patients (32.0%) scoring zero and 78 (19.5%) scoring 8–10. Cardiac troponin was above the upper reference limit at presentation or at one hour in 96 patients (24.0%): 3 of 264 low-risk patients (1.1%), 7 of 50 moderate-risk patients (14.0%) and all 86 high-risk patients (100%). Eighty-three patients (20.8%) were admitted to hospital, almost all from the high-risk category (82 of 86, 95.3%), compared with 1 of 50 moderate-risk patients (2.0%) and no low-risk patient. All 51 revascularisation procedures (46 percutaneous coronary interventions and 5 coronary artery bypass grafts) and all five in-hospital deaths (1.2% of the cohort) occurred among high-risk patients. In an exploratory, hypothesis-generating multivariable analysis of troponin elevation, older age (adjusted odds ratio 2.38 per decade, 95% CI 1.88–3.09) and smoking (4.10, 2.12–8.06) were associated with elevation and female sex was inversely so (0.22, 0.11–0.41); renal function was not available for adjustment. **Conclusions:** HEART score categories were associated with ED disposition and in-hospital management in this retrospective cohort of COVID-19 survivors presenting with acute chest pain. High-risk patients accounted for nearly all hospital admissions, coronary revascularizations, and in-hospital deaths. However, systematic post-discharge follow-up was not available; therefore, 30-day or 6-week MACE could not be assessed, and the prognostic performance of the HEART score or the safety of HEART-based discharge cannot be determined. These findings should therefore be interpreted as descriptive associations reflecting real-world clinical practice and warrant confirmation in prospective studies with systematic follow-up.

## 1. Introduction

Chest pain is one of the most common reasons patients present to the Emergency Department (ED), accounting for millions of healthcare encounters worldwide. Only a proportion of these patients are ultimately diagnosed with acute coronary syndrome (ACS), yet emergency clinicians still face the difficult task of separating those at risk for major adverse cardiovascular events (MACE) from those unlikely to have serious cardiac disease. Missing or delaying an ACS diagnosis can raise morbidity and mortality. At the same time, avoidable hospital admissions, extensive diagnostic testing, and prolonged ED observation add to overcrowding and rising healthcare costs [1,2].

Assessing acute chest pain can be especially difficult in patients with a history of SARS-CoV-2 infection [3,4,5,6]. After the acute illness, COVID-19 survivors may continue to have, or may newly develop, cardiovascular abnormalities such as myocardial injury, endothelial dysfunction, thrombo-inflammatory activation, autonomic disturbances, and a greater burden of cardiovascular risk factors [7,8]. As a result, acute chest pain may be harder to interpret, underscoring the need for risk stratification that is structured and reproducible.

Several clinical prediction models support early cardiovascular risk assessment, among them the Thrombolysis in Myocardial Infarction (TIMI) and Global Registry of Acute Coronary Events (GRACE) scores. Yet because these tools were developed mainly for patients with established or suspected ACS, their usefulness may be limited in the heterogeneous group presenting to the ED with undifferentiated chest pain [9].

In 2008, Six et al. introduced the HEART score, a bedside tool for stratifying risk in patients with acute chest pain [10]. Its name comes from five routinely available clinical variables: History, Electrocardiogram (ECG), Age, Risk factors, and Troponin. Each variable receives 0–2 points, producing a total score between 0 and 10. Based on the estimated probability of short-term adverse cardiac outcomes, patients are then classified as low, intermediate, or high risk [11].

Since its introduction, researchers have examined the HEART score across varied patient groups and healthcare settings. Prospective and retrospective studies have shown that it can distinguish patients by short-term cardiovascular risk and, when paired with appropriate clinical assessment and serial cardiac biomarker testing, help identify those who may be discharged early [12,13]. Among patients correctly classified as low risk, MACE occurs very infrequently. By contrast, those placed in the high-risk category generally need more extensive diagnostic evaluation and cardiovascular management [14].

Current international recommendations for patients presenting with acute chest pain call for structured clinical risk assessment alongside ECG evaluation and serial measurements of high-sensitivity cardiac troponin [15,16]. The HEART score continues to be appealing in this setting: it is simple, accessible, and based on clinical information that is routinely available. Used within accelerated diagnostic pathways, structured risk assessment may shorten ED stays and reduce unnecessary hospitalizations without compromising patient safety [17].

However, patients with prior SARS-CoV-2 infection may have clinical characteristics that differ from those seen in conventional chest-pain populations [18]. Cardiovascular symptoms in COVID-19 survivors can arise through several potential mechanisms [19], yet the suitability of established chest-pain risk scores for this group has not been adequately defined. The HEART score’s performance and clinical distribution may also differ with demographic characteristics, cardiovascular risk profiles, the organization of local healthcare, and clinical practice. Evidence on the use of the HEART score in patients presenting with acute chest pain remains limited in Romania [20]. To the best of our knowledge, no earlier study has specifically examined the distribution of the HEART score and its association with in-hospital management and outcomes among COVID-19 survivors presenting with acute chest pain in the ED.

The distribution of the HEART score and its association with ED disposition and in-hospital management and outcomes among COVID-19 survivors presenting to the ED with acute chest pain. The cohort comprised 400 consecutive patients treated in a tertiary emergency department. We assessed the distribution of individual HEART score components and overall risk categories, then examined their associations with cardiac troponin elevation, hospitalization, coronary revascularization, therapeutic management, and short-term in-hospital outcomes.

## 2. Materials and Methods

### 2.1. Study Design and Setting

This retrospective observational study took place in the Emergency Department of the Municipal Clinical Hospital of Timișoara, Romania. Eligibility screening included consecutive adults who presented with acute, non-traumatic chest pain from 1 June 2023 through 31 December 2024. More than 30,000 patients are assessed by the department each year, with laboratory testing and diagnostic imaging available continuously, 24 h a day. During the study period, its staff comprised 12 emergency medicine consultants and approximately 60 residents at varying stages of training. Patient evaluation and management followed the applicable national and international recommendations.

For the purposes of this study, “COVID-19 survivors” referred to adults with a documented previous SARS-CoV-2 infection who had survived the acute phase of COVID-19 and subsequently presented to the Emergency Department with acute, non-traumatic chest pain. This terminology is consistent with the use of the term “COVID-19 survivors” by the U.S. Centers for Disease Control and Prevention (CDC) to describe individuals who survived a previous COVID-19 episode [21]. No minimum interval from the previous COVID-19 episode to the index Emergency Department presentation was prespecified. The term “COVID-19 survivors” was used to describe survival following a previous COVID-19 episode and did not imply the presence of persistent symptoms, Long COVID, or post-COVID-19 condition. Patients were included regardless of the severity of the previous COVID-19 episode or whether hospitalization or intensive care had been required.

### 2.2. Study Population

Patients were included if they were aged ≥18 years, had a documented history of SARS-CoV-2 infection and survived the acute COVID-19 episode, and presented to the Emergency Department with acute, non-traumatic chest pain or discomfort that was considered potentially cardiac during the initial assessment. Complete clinical and laboratory records also had to be available so that all five HEART score components could be calculated retrospectively: clinical history, initial electrocardiogram (ECG), age, documented cardiovascular risk factors, and the cardiac troponin concentration at presentation.

Patients were excluded when they were under 18, when the chest pain had an apparent non-cardiac cause at presentation—such as chest trauma, pneumothorax, or pneumonia—or when the initial ECG showed ST-segment elevation myocardial infarction (STEMI). Exclusion also applied to patients transferred from another hospital with an established diagnosis of acute coronary syndrome (ACS), as well as those whose incomplete medical records made accurate HEART score calculation impossible.

Previous SARS-CoV-2 infection was established through documented laboratory confirmation in the medical record. COVID-19 survivors were defined as described in Section 2.1.

**Figure 1:**
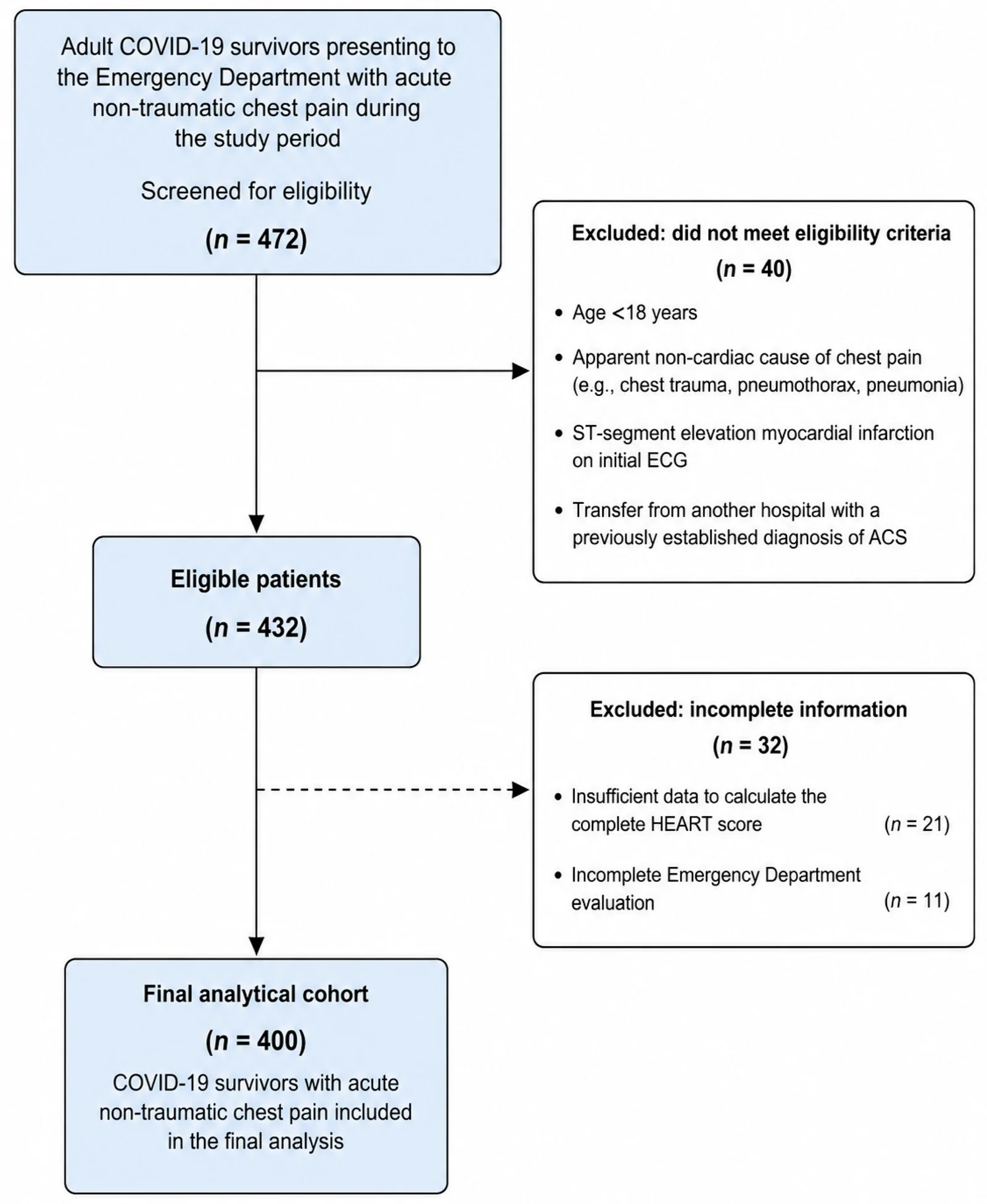
Flowchart of participant screening, eligibility assessment, and inclusion in the final analytical cohort.

During the study period, 472 consecutive adults who had survived COVID-19 and presented to the Emergency Department with acute, non-traumatic chest pain were screened. Forty failed to meet the predefined eligibility criteria, leaving 432 eligible patients. Another 32 were excluded because information needed for the analysis was incomplete: 21 lacked sufficient data to calculate the complete HEART score, and 11 had not undergone a complete Emergency Department evaluation. The final analytical cohort therefore comprised 400 patients. Figure 1 presents the participant selection process.

### 2.3. HEART Score Assessment

The HEART score [11] was not calculated or documented prospectively by the treating clinicians and was not used to guide clinical management. For the purposes of this retrospective study, the score was reconstructed from routinely available clinical, electrocardiographic, and laboratory data by two physicians independently. It includes five routinely available variables: History, Electrocardiogram (ECG), Age, Risk factors, and Troponin. Under the original HEART score criteria, each variable received 0, 1, or 2 points, producing a total score from 0 to 10. For History, scoring reflected how suspicious the presenting chest-pain characteristics were for myocardial ischemia. ECG scoring was based on the presence and extent of repolarization abnormalities, while Age was scored using the predefined HEART score age categories. Risk factors comprised established cardiovascular risks and documented atherosclerotic disease recorded in the medical record. The Troponin component was assigned a score based on the patient’s cardiac troponin concentration in relation to the upper reference limit applied by the local laboratory.

The scores for the individual components were added together to calculate each patient’s total HEART score. Patients were then placed into one of three predefined risk categories: low risk (0–3 points), moderate risk (4–6 points), or high risk (7–10 points).

### 2.4. Laboratory Testing

Cardiac troponin I was measured using the VIDAS^®^ High sensitive Troponin I (TNHS) assay (**bioMérieux** SA, Marcy l’Etoile, France) on the miniVIDAS immunoassay system, based on the enzyme-linked fluorescent assay (ELFA) principle. The assay is a high-sensitivity cardiac troponin I assay, with a reported limit of detection of 0.8 ng/L and limit of quantification of 3.2 ng/L. The manufacturer-reported overall 99th-percentile upper reference limit is 19 ng/L, with sex-specific 99th-percentile values of 25 ng/L for men and 11 ng/L for women. In the present study, however, the local laboratory’s validated upper reference limit was used for clinical interpretation and for determining the troponin component of the HEART score; sex-specific thresholds were not applied. Blood samples were collected at ED presentation in accordance with standard venipuncture procedures, centrifuged at 3000× *g* for 10 min, and analyzed immediately. For patients undergoing serial biomarker assessment, a second measurement was obtained 1 h after the first sample (0 h/1 h strategy). Results were interpreted according to the manufacturer’s instructions and the local laboratory’s validated cut-off values.

### 2.5. Outcomes

The primary outcomes were the distribution of HEART score risk categories and their relationship with ED disposition and in-hospital management. Secondary outcomes included cardiac troponin elevation at presentation or at one hour, hospital admission, length of hospital stay, conservative treatment, coronary revascularization, either by percutaneous coronary intervention (PCI) or coronary artery bypass grafting (CABG), and in-hospital mortality.

### 2.6. Statistical Analysis

All 400 patients were retained after the two selection stages shown in Figure 1. No values were missing for any of the 28 study variables, and no imputation was therefore performed. The analysis is therefore a complete-case analysis of the 400 patients (92.6% of the 432 eligible). The characteristics of the 32 patients excluded at that stage are not held in the analysis dataset, so no comparison between them and the patients analyzed is presented. Descriptive tables are stratified by HEART risk category (low, 0–3; moderate, 4–6; high, 7–10).

Analyses were performed in R version 4.6.0 (R Foundation for Statistical Computing, Vienna, Austria) using the packages readxl 1.5.0 for data import, dplyr 1.2.1 for data manipulation, DescTools 0.99.60 for exact binomial confidence intervals, effsize 0.8.1 for Cliff’s delta, and ggplot2 4.0.3 for the figures. We obtained profile-likelihood confidence intervals with the confint method for generalized linear models in the stats package.

We examined the distribution of each continuous variable graphically and by calculating skewness. Because cardiac troponin concentrations and length of hospital stay were markedly right-skewed, we summarised all continuous variables as the median with the interquartile range (IQR). We report categorical variables as absolute frequencies and percentages. The three-level components of the HEART score are ordinal and are reported as frequencies and percentages of patients at each level.

Formal comparisons were limited to characteristics associated with elevated cardiac troponin concentrations. Continuous variables were compared between the two troponin groups by the Mann–Whitney *U* test. Categorical variables were compared by Pearson’s chi-squared test without continuity correction. Fisher’s exact test was pre-specified for any table in which an expected cell count fell below five; in the event, the smallest expected count was 5.3, and the chi-squared test was therefore used throughout. All tests were two-sided, and all confidence intervals are at the 95% level. No threshold of statistical significance was applied: the study is descriptive, its *p*-values are interpreted as descriptive quantities.

Effect sizes accompany these comparisons and carry more interpretative weight than *p*-values. For continuous variables, we report Cliff’s delta with its 95% confidence interval. For categorical variables, we report both the risk ratio and the odds ratio, each with a 95% confidence interval. The outcome occurs in 24.0% of the cohort, well above the frequency at which the odds ratio approximates the risk ratio, and reporting the odds ratio alone would materially overstate the association.

Where the association between two ordinal clinical variables is described, Spearman’s rank correlation is used, with a confidence interval obtained by Fisher’s z transformation.

The number of patients with an elevated troponin concentration and the number discharged from the emergency department with one both depend on the threshold applied; we therefore recomputed both counts at six thresholds spanning about seven-tenths to about one and eight-tenths of the upper reference limit, which is itself among them. We chose the thresholds after inspecting the distribution, so this analysis is post hoc and exploratory; we report the counts descriptively, with no test of trend, in Supplementary Table S2.

A single multivariable logistic regression model was fitted, with a cardiac troponin concentration above the upper reference limit at either time point as the dependent variable. This analysis is secondary to the principal objective of the study; it is explicitly exploratory and hypothesis-generating, and was fitted to describe adjusted associations, with no causal interpretation intended. Covariates were specified in advance and restricted to objectively documented patient characteristics—age, sex, arterial hypertension, diabetes mellitus, smoking and known atherosclerotic disease—none of which is derived from the troponin measurement. We chose them on clinical grounds, as characteristics established as determinants of myocardial injury, not according to their univariable *p*-values; we applied no variable-selection procedure. With 96 events and six covariates, the ratio of events per variable was 16.0. We examined model assumptions (linearity of age on the logit scale, variance inflation factors, the Hosmer–Lemeshow test, and Cook’s distance); results are reported in Supplementary Methods S1. Adjusted odds ratios are reported with profile-likelihood confidence intervals and likelihood-ratio *p*-values. Renal function was not recorded in the registry and could not be included; because impaired renal function is a principal determinant of chronic troponin elevation, the model is unadjusted for it, and its estimates must be interpreted accordingly. The odds ratio for age is expressed per ten years.

Because renal function could not be measured, the sensitivity of the adjusted estimates to an unmeasured confounder was quantified by the E-value, computed from adjusted risk ratios obtained by marginal standardisation of the fitted model, with nonparametric bootstrap confidence intervals. The method and its results are reported in Supplementary Methods S1 and Supplementary Table S1. Because the E-value is defined for a causal contrast whereas this model estimates adjusted associations, it is presented only as a supplementary illustration of how fragile a causal reading would be, and not as a basis for causal inference.

Proportions of principal interest are reported with exact (Clopper–Pearson) 95% confidence intervals, preferred to normal-approximation intervals because several subgroups contained few events.

No measure of discrimination or predictive accuracy is reported—no receiver operating characteristic analysis, sensitivity, specificity or predictive value—because cardiac troponin and the electrocardiogram contribute both to the HEART score and to the decisions that determined admission and treatment; any such estimate would reflect that shared input rather than predictive performance. For the same reason, we did not fit a multivariable model of admission or treatment, nor one for in-hospital mortality, since five deaths do not support an adjusted analysis.

Neither *p*-values nor effect sizes appear in the tables stratified by HEART risk category. Those categories are defined by the total score, which includes age, individual cardiovascular risk factors, and troponin concentration, so any measure of association between them is determined by the score’s construction rather than observed.

This study is descriptive and did not test a pre-specified primary hypothesis; the analysis plan was not pre-registered. We report 12 formal univariate comparisons and one multivariable model, all within a single section of the Results; at a two-sided threshold of 0.05, approximately 0.6 nominally significant results would be expected by chance alone. No adjustment for multiplicity was applied, and all *p*-values should therefore be read as descriptive quantities rather than as formal tests. Exact *p*-values are reported to three decimal places, with values below 0.001 reported as *p* < 0.001.

## 3. Results

### 3.1. Baseline Characteristics

Table 1 presents the characteristics of the COVID-19 survivors cohort, overall and by HEART risk category. The median age was 52.5 years (IQR 36.0–66.0) and 209 patients (52.2%) were male. One hundred fifty-one patients (37.8%) were from rural areas. Median systolic and diastolic blood pressures at presentation were 145 mmHg (IQR 130–160) and 80 mmHg (IQR 70–90).

Arterial hypertension was the most frequent cardiovascular risk factor, recorded in 155 patients (38.8%), followed by smoking in 81 (20.2%), diabetes mellitus in 58 (14.5%), and obesity in 57 (14.2%). A positive family history was documented in 52 patients (13.0%), known atherosclerotic disease in 26 (6.5%), and hypercholesterolemia in 22 (5.5%). The median number of risk factors among the six, as counted by the score, was 1 (IQR 0–2).

Age and comorbidity burden increased across the three risk categories: the median age was 42.0 years in the low-risk category, 67.5 years in the moderate-risk category, and 69.0 years in the high-risk category, and the median number of risk factors increased from 0 to 2 (Table 1). We did not test these differences statistically because the HEART score defines the categories, and age and risk factors are components of the score; any such comparison would reflect the score’s construction rather than observation.

### 3.2. Distribution of the HEART Score

**Figure 2:**
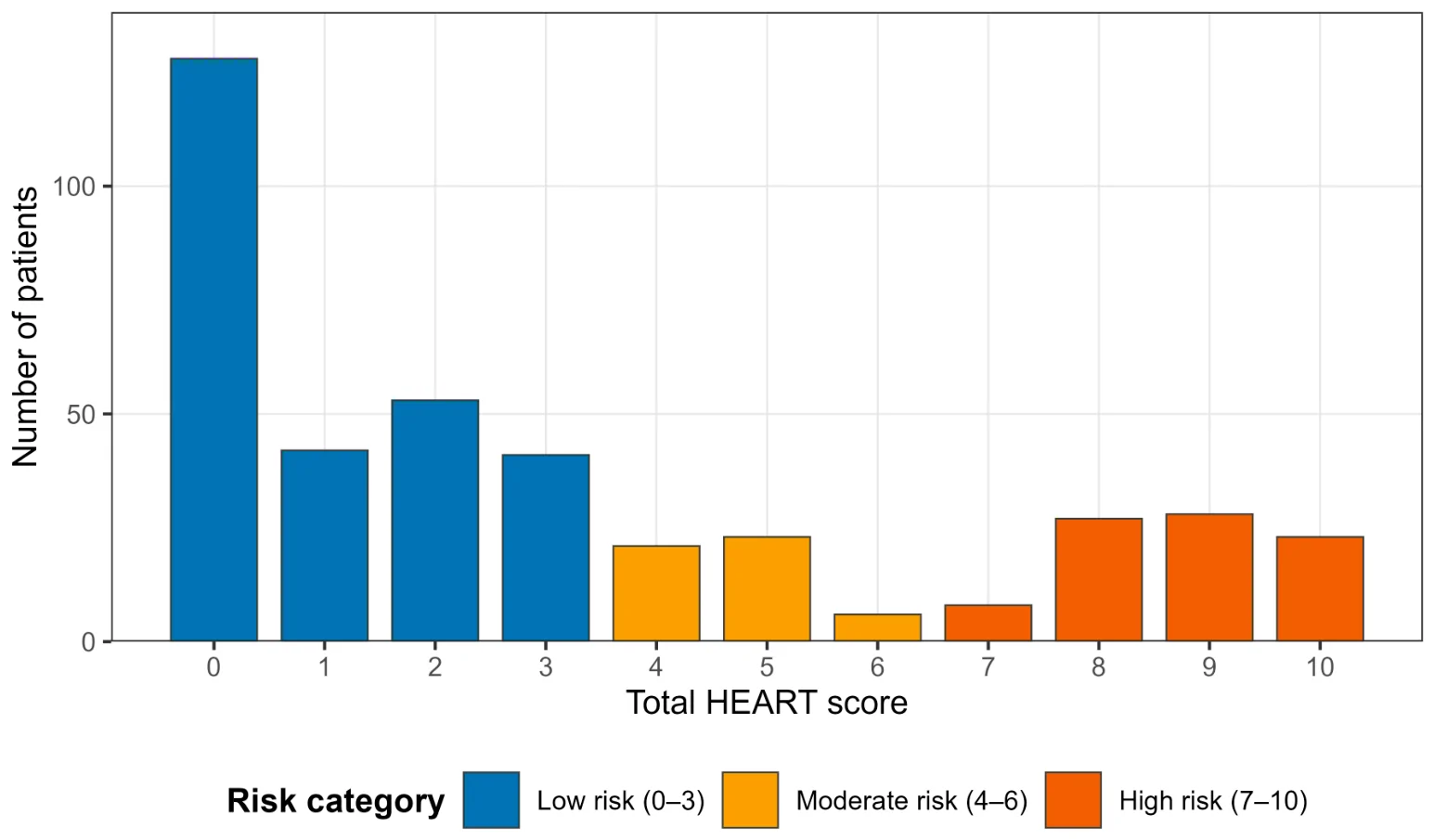
Distribution of the total HEART score in all 400 patients. The horizontal axis gives the total score, from 0 to 10, and the vertical axis the number of patients with each score; bars are coloured by risk category (low, 0–3; moderate, 4–6; high, 7–10).

The distribution of the five components and of the total score is given in Table 2, and the distribution of the total score is shown in Figure 2. The median total score was 2 (IQR 0–5). 264 patients (66.0%) fell into the low-risk category (score 0–3), 50 (12.5%) into the moderate-risk category (4–6), and 86 (21.5%) into the high-risk category (7–10).

The total score distribution was bimodal (Figure 2). The most frequent value was zero, recorded in 128 patients (32.0%), followed by a smaller group at scores of 8 to 10 (78 patients, 19.5%). Six patients (1.5%) had a score of 6 and eight (2.0%) a score of 7.

The history was graded as slightly suspicious in 279 patients (69.8%), moderately suspicious in 37 (9.2%) and highly suspicious in 84 (21.0%). The electrocardiogram was normal in 270 patients (67.5%), showed a non-specific repolarisation abnormality in 41 (10.2%), and significant ST-segment deviation in 89 (22.2%).

The two components graded by clinical judgment were closely associated. Among the 86 patients in the high-risk category, 79 (91.9%) had both a highly suspicious history and significant ST-segment deviation; across the whole cohort, the rank correlation between the two gradings was 0.799 (95% CI 0.761–0.832).

### 3.3. Cardiac Troponin at Presentation and at One Hour

**Figure 3:**
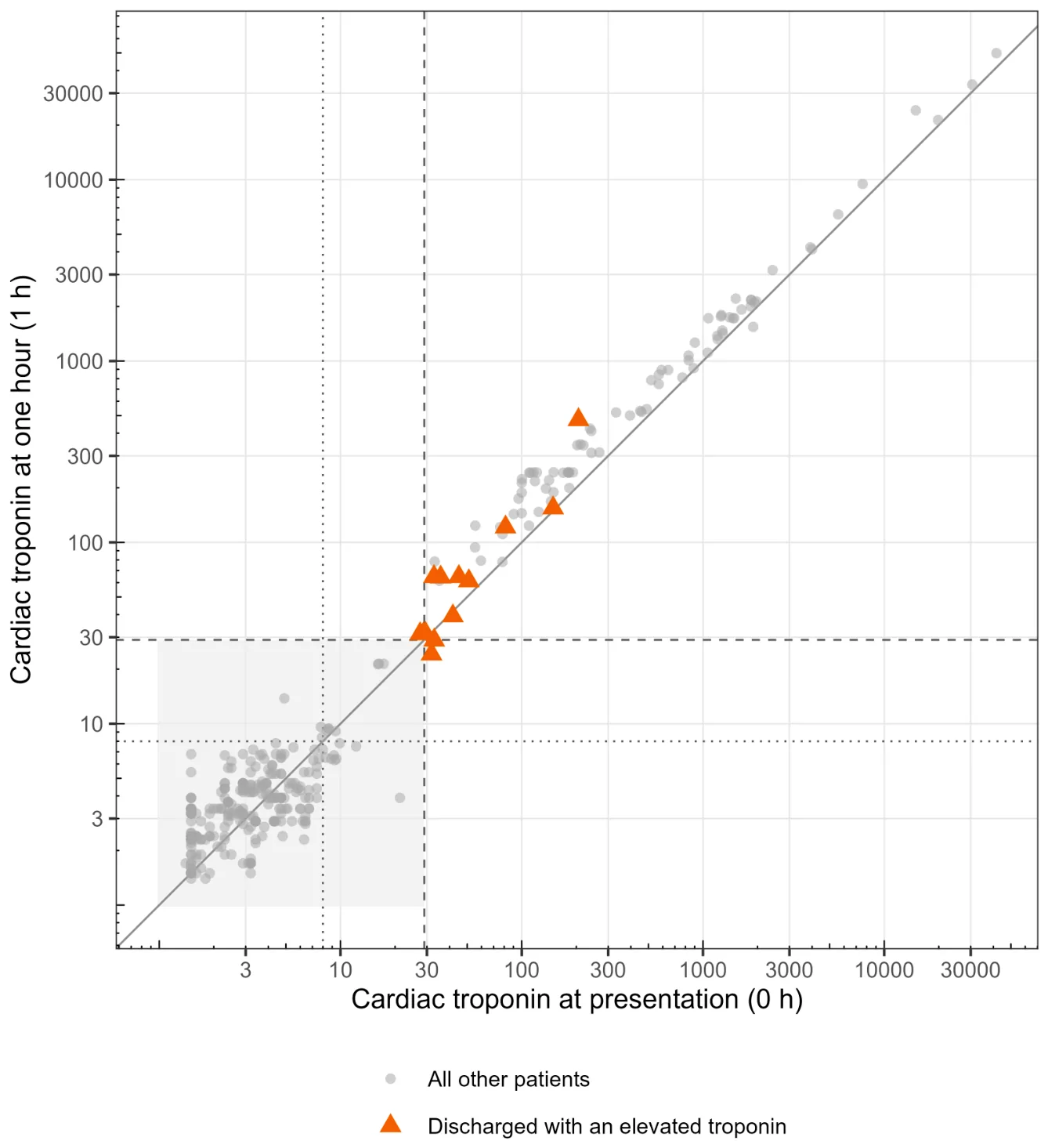
Cardiac troponin at presentation and one hour later in all 400 patients, on logarithmic axes. Dotted lines mark the lower value of the laboratory reference interval and dashed lines the upper reference limit; the solid diagonal is the line of identity and the shaded square the region in which both measurements lie at or below the upper reference limit. Triangles denote the 13 patients discharged from the emergency department with a concentration above the upper reference limit at one or both time points (listed individually in Supplementary Table S3). The interval between the two samples was fixed at one hour by protocol. Both axes are in ng/L. All concentrations are plotted as reported by the laboratory; no value was censored, substituted, or imputed.

The distribution of both troponin measurements, expressed in relation to the laboratory reference interval, is given in Table 3, and the relationship between the two is shown in Figure 3. All troponin concentrations are reported in ng/L; the laboratory reference interval is 8–29, and the ULN is therefore 29. The lowest concentration recorded at either time point was 1.4, and 83 presentation values (20.8%) were exactly 1.5.

At presentation, 306 measurements (76.5%) were at or below the ULN and 94 (23.5%) were above it; at one hour, the figures were the same. Of those at or below the limit, 288 (72.0% of the cohort) at presentation and 294 (73.5%) at one hour lay below the lower value of the interval reported by the laboratory. Those patients are not distinguishable from the remainder of the sub-threshold group for any purpose of the score. Ninety-six patients (24.0%) had a concentration above the ULN at one or both time points: three of 264 patients (1.1%) in the low-risk category, seven of 50 (14.0%) in the moderate-risk category and all 86 (100%) in the high-risk category. The last of these is not a necessary consequence of the way the score is constructed, since a total of seven or eight points is attainable without any contribution from troponin; 53 patients reached seven or more points from the other four components alone, and all of them also had a troponin concentration above the ULN.

Status relative to the ULN changed between the two measurements in four patients. Of the 306 patients whose presentation value was at or below the limit, two (0.65%; 95% CI 0.08–2.34) exceeded it at 1 h, one newly identified elevation for approximately every 153 s measurements performed. Conversely, of the 94 patients whose presentation value was above the limit, two (2.13%; 95% CI 0.26–7.48) had fallen to or below it by one hour.

Among the 94 patients whose presentation concentration exceeded the ULN, the median one-hour-to-presentation ratio was 1.32 (IQR 1.15–1.59), and the concentration had risen in 89 (94.68%; 95% CI 88.02–98.25). The two measurements were closely correlated in this subgroup (Spearman ρ = 0.985, 95% CI 0.977–0.990). The local protocol fixed the interval between the two samples at 1 h, so these changes reflect a uniform observation window; however, the time since symptom onset was not recorded, so they cannot be related to the stage of the patient’s presentation.

### 3.4. Disposition and In-Hospital Course

Eighty-three patients (20.8%) were admitted to hospital, and 317 (79.2%) were discharged directly from the emergency department (Table 4). Admission was confined almost entirely to the high-risk category, from which 82 of 86 patients (95.3%) were admitted; 1 of 50 patients in the moderate-risk category (2.0%) and none of 264 in the low-risk category were admitted.

Among the 83 admitted patients, the median length of stay was 4.0 days (IQR 3.0–5.5). Thirty-two patients (8.0% of the cohort) received conservative medical therapy, 46 (11.5%) underwent percutaneous coronary intervention, and 5 (1.2%) underwent coronary artery bypass grafting, for a total of 51 revascularisation procedures (12.8%). The remaining 317 patients (79.2%) received no treatment during the index episode.

Five patients (1.2%) died in hospital, all of them in the high-risk category (5.8% of that category). No follow-up after discharge was undertaken, so no events occurring beyond the index episode are reported.

### 3.5. Characteristics Associated with an Elevated Troponin Concentration

Ninety-six patients (24.0%) had an elevated troponin concentration at one or both time points. Table 5 presents the univariate comparisons, and Table 6 presents the multivariable model, which is secondary to the principal objective of the study and is reported as an exploratory, hypothesis-generating analysis. Twelve univariate comparisons are reported; ten reached nominal significance, against approximately 0.6 expected by chance at a two-sided threshold of 0.05.

Patients with an elevated concentration were substantially older than those without (median 68.0 years, IQR 57.0–75.2, versus 46 years, IQR 33–62; Cliff’s delta +0.621, 95% CI +0.524 to +0.702, a large effect; *p* < 0.001). Systolic blood pressure was higher in the elevated group (median 150 versus 140 mmHg; *p* = 0.047), but the effect size was negligible, and its confidence interval included zero (Cliff’s delta +0.134, −0.013 to +0.276), so this difference is not robust. For diastolic pressure, the effect size was likewise negligible, and its interval included zero (+0.106, −0.035 to +0.242; *p* = 0.115).

All seven cardiovascular conditions included by the score were associated with elevated concentrations. Because the outcome occurs in 24.0% of the cohort, risk ratios are preferred to odds ratios, which at this frequency overstate the association considerably; both are shown in Table 5. The strongest univariate associations were with known atherosclerotic disease (risk ratio 3.55, 95% CI 2.61–4.82; odds ratio 10.47, 4.25–25.81), arterial hypertension (3.16, 2.18–4.59), smoking (2.94, 2.13–4.05), hypercholesterolaemia (2.93, 2.03–4.24) and diabetes mellitus (2.55, 1.83–3.57). Female sex was associated with a lower frequency of elevation (29 of 191 women, 15.2%, versus 67 of 209 men, 32.1%; risk ratio 0.47, 0.32–0.70). Area of residence was the only categorical characteristic whose interval included the null (23.2% of rural and 24.5% of urban patients; risk ratio 0.95, 0.66–1.36; *p* = 0.765), that interval remaining compatible with a risk about a third higher or a third lower.

We fitted a multivariable model with 96 events and six covariates (16.0 events per variable) to describe how these characteristics interact (Table 6). This analysis is exploratory and hypothesis-generating; the estimates describe adjusted associations and no causal interpretation is intended. Age (adjusted odds ratio 2.38 per decade, 95% CI 1.88–3.09; *p* < 0.001) and smoking (4.10, 2.12–8.06; *p* < 0.001) showed adjusted associations with an elevated concentration, and female sex an inverse one (0.22, 0.11–0.41; *p* < 0.001). The estimate for known atherosclerotic disease is imprecise (2.95, 1.02–9.27; *p* = 0.046), rests on 26 patients and spans almost an order of magnitude; the corresponding standardized risk ratio is 1.59, with a bootstrap confidence interval of 0.88–2.63 that includes the null. It should not be read as a firm result. After mutual adjustment, the estimates for arterial hypertension (1.58, 0.86–2.94; *p* = 0.144) and diabetes mellitus (1.57, 0.77–3.21; *p* = 0.210) were imprecise, their confidence intervals including the null while extending to odds roughly three times as high; neither the absence of an association nor a substantial one can be excluded. Renal function was not recorded and could not be entered into the model; because impaired renal function is a principal determinant of chronic troponin elevation, these adjusted estimates describe the observed associations rather than establish independence.

As a supplementary sensitivity analysis, the robustness of these estimates to unmeasured confounding—in particular to the absence of renal function—was quantified by the E-value, computed from adjusted risk ratios obtained by standardising the fitted model over the cohort. The method and the resulting values are reported in Supplementary Methods S1 and Supplementary Table S1. They indicate how fragile a causal reading of these estimates would be; they do not rule out residual confounding and do not support a causal interpretation.

Model assumptions were examined, and none was violated. Age was linear on the logit scale (Box–Tidwell *p* = 0.796), there was no material collinearity (all variance inflation factors ≤ 1.42), and no observation was influential (maximum Cook’s distance 0.059). The Hosmer–Lemeshow test did not detect a departure from calibration (*p* = 0.277), a test of limited power. Full details are given in Supplementary Methods S1.

## 4. Discussion

The present study examined the distribution of the HEART score and its association with ED disposition and in-hospital management and outcomes in 400 consecutive COVID-19 survivors presenting to the Emergency Department with acute, non-traumatic chest pain. Patients separated clearly by HEART risk category: 66.0% were classified as low risk, 12.5% as moderate risk, and 21.5% as high risk. The differences extended to hospital disposition and management. No low-risk patients were admitted or underwent coronary revascularization, while 95.3% of high-risk patients were admitted and 59.3% underwent PCI or CABG. Elevated cardiac troponin was likewise concentrated among patients with higher HEART scores. The findings add to the evidence on HEART-based chest-pain assessment in a population that remains understudied: patients with a documented history of COVID-19.

### 4.1. HEART Score Distribution in COVID-19 Survivors

The HEART score was originally developed as a simple bedside tool integrating five routinely available variables—History, ECG, Age, Risk factors, and Troponin—to facilitate structured risk assessment in patients presenting with chest pain. Subsequent prospective validation studies and systematic reviews have demonstrated increasing rates of adverse cardiac outcomes with increasing HEART scores and have supported the identification of low-risk patients who may be candidates for early discharge when the score is incorporated into an appropriate diagnostic pathway [15,22,23,24].

In the present cohort, the median HEART score was 2 (IQR 0–5), with approximately two-thirds of patients classified as low risk. Interestingly, the distribution was bimodal, with 32.0% of patients having a score of 0 and 19.5% having scores of 8–10. This pattern suggests the presence of two clinically distinct groups within the study population: a large group with few features suggestive of acute coronary pathology and a smaller group with a substantial accumulation of clinical, electrocardiographic, age-related, and biomarker abnormalities.

The distribution should nevertheless be interpreted in the context of the specific study population. The HEART score was developed and validated in heterogeneous emergency-department chest-pain populations, whereas COVID-19 survivors may have different cardiovascular characteristics, comorbidity profiles, referral patterns, and mechanisms of chest pain. Previous studies have documented persistent cardiovascular abnormalities after COVID-19, including myocardial injury, endothelial dysfunction, thrombo-inflammatory activation, and longer-term cardiovascular complications [7,8,12,13]. Thus, the bimodal distribution observed in our cohort should not be interpreted as evidence of altered HEART score performance but rather as a reflection of the characteristics of this particular emergency population.

### 4.2. Relationship Between HEART Score and Hospital Disposition

A particularly clinically relevant finding was the marked difference in hospital admissions across HEART categories: no low-risk patients were admitted, whereas admission occurred in 1 of 50 moderate-risk patients and 82 of 86 high-risk patients. The same pattern appeared for coronary revascularization. All 51 procedures—46 PCI and 5 CABG—were performed in the high-risk group, which also accounted for all five in-hospital deaths.

These findings fit the HEART score’s intended clinical purpose: as patients accumulate more high-risk features, they are more likely to need intensive diagnostic assessment and treatment. Earlier studies indicate that HEART-based accelerated diagnostic pathways can reduce unnecessary hospital admissions and cardiac testing while preserving a low rate of adverse outcomes in appropriately selected low-risk patients [6,22,24,25]. In particular, randomized and implementation studies of the HEART Pathway have demonstrated reductions in hospitalization and objective cardiac testing, with very low rates of death or myocardial infarction among patients classified as low risk [22,26].

However, the present findings should not be interpreted as prospective validation of the HEART score or as evidence that HEART-guided discharge is safe in COVID-19 survivors. The score includes ECG abnormalities and cardiac troponin, both of which directly influence clinicians’ decisions regarding admission, coronary angiography, and revascularization. Consequently, part of the observed association between HEART category and management is expected to arise from the overlap between the score components and the information used in clinical decision-making.

This issue matters especially for COVID-19 survivors, whose chest pain may arise through several cardiovascular and non-cardiovascular mechanisms. Current guidelines state that structured risk assessment should supplement—not replace—clinical judgment, ECG interpretation, and serial cardiac troponin assessment [8,10,15,16]. Therefore, the present findings support the clinical usefulness of HEART as an organizing framework, but not its use as an autonomous discharge decision rule.

### 4.3. Cardiac Troponin and HEART Risk Stratification

Cardiac troponin elevation was observed in 96 patients (24.0%) at either presentation or 1 h. The concentration of elevated troponin across HEART categories was striking: only 3 of 264 low-risk patients (1.1%) had an elevated concentration, compared with 7 of 50 moderate-risk patients (14.0%) and all 86 high-risk patients (100%). This finding needs careful interpretation: troponin is one of the five components of the HEART score. Therefore, the higher frequency of troponin elevation in higher HEART categories is partly expected by the construction of the score and should not be interpreted as an independent association or as evidence of independent predictive performance. The strong association between the total HEART score and troponin elevation is therefore partly built into the score itself, so it cannot be viewed as an independent association. Still, the 1 h troponin measurement was not used to calculate the HEART score and thus supplied additional information about biomarker changes after the initial assessment.

Serial troponin testing aligns with current recommendations for patients suspected of having ACS [10,15,16]. In our cohort, only four patients crossed the laboratory ULN between presentation and 1 h. Among patients with an elevated initial concentration, 89 of 94 (94.7%) demonstrated a subsequent increase, and the two measurements were strongly correlated (Spearman ρ = 0.985). Nevertheless, the time from symptom onset was unavailable, preventing interpretation of these changes according to the biological stage of myocardial injury.

An important clinical distinction is that troponin elevation indicates myocardial injury but does not, by itself, establish acute myocardial infarction. According to the Fourth Universal Definition of Myocardial Infarction, myocardial infarction requires evidence of acute myocardial injury together with clinical evidence of myocardial ischemia. Troponin elevation may occur in numerous cardiac and non-cardiac conditions, including heart failure, tachyarrhythmia, hypoxemia, shock, myocarditis, and renal dysfunction [23].

This distinction is particularly relevant to the 13 patients who were discharged despite elevated troponin at one or both time points, whose individual characteristics are listed in Supplementary Table S3. Their management cannot be judged as safe or unsafe from the present dataset because post-discharge outcomes were unavailable. Rather, these patients illustrate the importance of integrating troponin with symptoms, ECG findings, serial measurements, cardiovascular risk factors, and alternative causes of myocardial injury [15,27].

### 4.4. Factors Associated with Troponin Elevation

Patients with elevated troponin were substantially older than patients without elevation: the median ages were 68.0 and 46 years, respectively. On univariable analysis, elevated troponin was also associated with hypertension, diabetes mellitus, obesity, hypercholesterolemia, smoking, a positive family history, and known atherosclerotic disease. In an exploratory, hypothesis-generating multivariable analysis, age and smoking showed the strongest positive adjusted associations; these estimates describe associations and do not establish causal effects. For every 10-year increase in age, the adjusted OR was 2.38 (95% CI 1.88–3.09); for smoking, it was 4.10 (95% CI 2.12–8.06). Female sex, by contrast, was inversely associated with troponin elevation (adjusted OR 0.22, 95% CI 0.11–0.41). The association with age is clinically plausible: older age brings a greater burden of coronary atherosclerosis and structural cardiovascular disease. Smoking showed a strong association as well, consistent with the established links between tobacco exposure, endothelial dysfunction, atherosclerosis, thrombosis, and myocardial injury.

The inverse association observed for female sex warrants particular caution. Circulating cardiac troponin concentrations differ by sex, and assay-specific 99th-percentile thresholds are well recognized. Lee et al. showed that applying sex-specific thresholds can substantially increase detection of myocardial injury and myocardial infarction in women. In their multicenter study, sex-specific high-sensitivity cardiac troponin I thresholds increased the identification of myocardial injury by 42% in women and 6% in men [24]. For that reason, the inverse association between female sex and troponin elevation in our cohort should not be taken as evidence that female sex is protective. Differences in troponin biology, assay characteristics, and the use of a common laboratory threshold instead of sex-specific reference limits may partly explain the finding. The strong association between smoking and elevated troponin is also clinically plausible. In our cohort, smoking remained associated with elevated cardiac troponin after adjustment (adjusted OR 4.10, 95% CI 2.12–8.06). The possibility that smoking status affects cardiac troponin concentrations offers a plausible biological context for this observation [27].

Accordingly, the lower frequency of troponin elevation observed among women in the present study should not be interpreted as evidence of a protective effect of female sex. It may partly reflect biological differences, assay characteristics, or the use of a common laboratory threshold rather than sex-specific reference limits.

### 4.5. Clinical Implications for COVID-19 Survivors

These findings may have practical relevance in clinical care. After COVID-19, chest pain can arise from several causes, such as ischemic heart disease, myocardial injury, myocarditis, thromboembolic disease, pulmonary complications, and other post-infectious cardiovascular manifestations [7,8,12,13]. Cardiac troponin elevation should therefore be interpreted in its appropriate clinical context. An elevated troponin concentration indicates myocardial injury but does not, by itself, establish a diagnosis of acute myocardial infarction. Myocardial injury may occur in both ischemic and non-ischemic conditions, including myocarditis and other cardiac or systemic disorders [23,27]. Therefore, elevated troponin or a high HEART score should not be interpreted in isolation, and clinical assessment should integrate symptoms, ECG findings, serial troponin measurements, cardiovascular risk factors, and alternative causes of myocardial injury.

With so many possible explanations, assessing patients on the basis of symptoms alone can be difficult. Respiratory comorbidities and functional impairment add another layer of uncertainty when cardiopulmonary symptoms are interpreted. Research in patients with chronic obstructive pulmonary disease has shown that more severe disease is linked to reduced functional capacity and poorer psychological and health-related outcomes. That evidence underscores the need to consider the wider cardiopulmonary context when evaluating patients with chest symptoms [28].

Nevertheless, the HEART score is best used to support clinical decisions, not replace clinical judgment. A low score should not outweigh clinically significant findings pointing to other serious diagnoses, and an elevated troponin should not automatically be interpreted as ACS. This matters especially in COVID-19 survivors, since prior SARS-CoV-2 infection may be linked to persistent cardiovascular abnormalities and non-ischemic causes of myocardial injury [6,7,8,13]. Digital emergency-department platforms may facilitate future implementation of standardized HEART-based assessment through automated score calculation, integration of serial troponin measurements, and structured clinical reassessment [29]. Future studies should determine whether such tools can support consistent implementation of HEART-based pathways without replacing clinical judgment.

Thus, our findings provide a rationale for evaluating a standardized HEART-based accelerated diagnostic pathway specifically in COVID-19 survivors, rather than assuming that evidence derived from general chest-pain populations automatically applies to this population.

### 4.6. Strengths and Limitations

Several features strengthen the study. Although HEART scores were independently calculated retrospectively by two physicians, formal interobserver agreement was not assessed. This represents a methodological limitation, particularly for the more subjective components of the HEART score, such as clinical history and ECG interpretation.

Several limitations deserve attention. Because the study was retrospective and conducted at a single center, its findings may not generalize broadly, and selection and information bias remain possible. The study reflects routine clinical practice in a Romanian Emergency Department, where evidence on applying the HEART score remains limited. Two physicians reconstructed the HEART scores independently, but retrospectively evaluating the History and ECG components could still have introduced classification bias. Formal interobserver agreement was not assessed, representing a methodological limitation, particularly for the more subjective components of the HEART score. Also, 32 otherwise eligible patients were excluded because complete HEART-score or ED-evaluation information was unavailable; their characteristics could not be compared with those of the included cohort.

The main limitation is that patients were not followed systematically after discharge. As a result, 30-day or 6-week MACE could not be assessed, so this study cannot determine the HEART score’s prognostic performance or whether HEART-based discharge is safe. That matters because previous studies supporting HEART-guided discharge included systematic follow-up [30].

An important interpretative consideration is that electrocardiographic abnormalities and cardiac troponin are themselves components of the HEART score and are also major determinants of subsequent clinical management, including hospital admission, coronary angiography, and revascularization. Therefore, the observed association between higher HEART categories and these management outcomes is partly expected from the construction of the score and from the clinical decision-making process. These findings should not be interpreted as demonstrating an independent effect of the HEART score on admission, angiography, or revascularization, or as evidence of causal predictive performance. Rather, they describe the relationship between retrospectively reconstructed HEART categories and management decisions in routine clinical practice.

Another important limitation is that the present study did not systematically adjudicate the final diagnosis underlying each episode of chest pain. Although hospital admission, coronary revascularization, and in-hospital mortality provide clinically relevant information on the management and short-term course of the cohort, they should not be interpreted as direct diagnostic endpoints for acute coronary syndrome or myocardial infarction. This distinction is particularly important because cardiac troponin elevation indicates myocardial injury but does not, by itself, establish myocardial infarction, and myocardial injury may occur in both ischemic and non-ischemic conditions, including myocarditis and other cardiac or systemic disorders [23,27]. Future prospective studies should therefore include systematic diagnostic adjudication of acute coronary syndrome, myocardial infarction, myocarditis, pulmonary embolism, and other clinically relevant causes of chest pain.

Other limitations included limited information on the interval between symptom onset and troponin measurement, making serial troponin kinetics difficult to interpret, and the absence of renal-function data, which may represent an important source of residual confounding in the analysis of elevated troponin. The multivariable analysis of troponin elevation is secondary to the principal objective of this study and is exploratory and hypothesis-generating; its estimates describe adjusted associations, should not be read as causal, and require confirmation in prospective cohorts. Future prospective studies should systematically collect renal-function parameters and incorporate them into adequately powered analyses of troponin elevation. The moderate-risk group was relatively small (n = 50), while the low number of in-hospital deaths (n = 5) prevented meaningful adjusted mortality modeling. Management also was not standardized under a prospectively implemented HEART protocol. Thus, the observed association between HEART category and admission or treatment reflects real-world clinical practice, not the effect of a HEART-guided intervention.

### 4.7. Future Research

Future studies should prospectively evaluate the HEART score in larger, multicenter cohorts of COVID-19 survivors presenting with acute chest pain. Such studies should incorporate sex-specific troponin thresholds, renal-function assessment, documentation of symptom onset, and systematic follow-up for 30-day and 6-week MACE.

Attention should be given to evaluating whether a HEART-based accelerated diagnostic pathway can safely reduce unnecessary hospitalization and diagnostic testing in COVID-19 survivors while maintaining appropriate sensitivity for ACS and other serious cardiovascular conditions. Multicenter studies in Romanian Emergency Departments would also help determine the generalizability of these findings and establish whether the observed distribution of HEART risk categories is reproducible across different healthcare settings.

## 5. Conclusions

In this single-center retrospective cohort of COVID-19 survivors who presented to the Emergency Department with acute, non-traumatic chest pain, HEART score categories distinguished clinically different groups, particularly in terms of ED disposition and in-hospital management. About two-thirds of the patients (66.0%) fell into the low-risk category. High-risk patients, by contrast, accounted for nearly all hospital admissions, all coronary revascularization procedures, and all in-hospital deaths.

Cardiac troponin elevation occurred mainly in patients with higher HEART scores, though a small subgroup with elevated troponin was discharged directly from the ED. An exploratory, hypothesis-generating multivariable analysis found positive adjusted associations between troponin elevation and both older age and smoking, and an inverse association for female sex; these are associations, not causal effects. Caution is warranted in interpreting these results, since renal function—an important determinant of troponin elevation—was unavailable for adjustment.

Overall, these findings indicate that the HEART score could offer a simple, structured way to initially assess acute chest pain in COVID-19 survivors by combining clinical history, ECG findings, age, cardiovascular risk factors, and cardiac troponin. It should still be used to support—not replace—clinical judgment, especially because chest pain in COVID-19 survivors can arise from ischemic or non-ischemic cardiovascular causes, thromboembolic disease, pulmonary conditions, or post-infectious mechanisms. These results also warrant further evaluation of a standardized HEART-based accelerated diagnostic pathway that includes serial high-sensitivity troponin assessment and clinical reassessment specifically for COVID-19 survivors.

Future multicenter prospective studies should evaluate HEART-based pathways in COVID-19 survivors using standardized serial high-sensitivity troponin measurements, appropriate sex-specific reference thresholds, renal function assessment, documented symptom onset, and systematic follow-up at 30 days and 6 weeks for MACE. Such studies should also determine whether a COVID-19-specific HEART-based accelerated diagnostic pathway can safely reduce unnecessary hospitalizations and diagnostic testing without compromising the detection of ACS or other serious cardiovascular conditions.

## Figures and Tables

**Table 1:** Baseline characteristics of the cohort, overall and by HEART risk category.

Characteristic	Total (n = 400)	Low Risk 0–3 (n = 264)	Moderate Risk 4–6 (n = 50)	High Risk 7–10 (n = 86)
Age, years	52.5 [36.0–66.0]	42.0 [32.0–55.0]	67.5 [65.0–73.8]	69.0 [58.2–76.0]
Male sex	209 (52.2)	130 (49.2)	19 (38.0)	60 (69.8)
Rural residence	151 (37.8)	95 (36.0)	23 (46.0)	33 (38.4)
Systolic blood pressure, mmHg	145 [130–160]	140 [130–155]	150 [130–160]	150 [130–165]
Diastolic blood pressure, mmHg	80 [70–90]	80 [70–90]	80 [70–85]	80.5 [73.5–90.0]
***Cardiovascular risk factors***				
Arterial hypertension	155 (38.8)	61 (23.1)	36 (72.0)	58 (67.4)
Diabetes mellitus	58 (14.5)	17 (6.4)	13 (26.0)	28 (32.6)
Obesity	57 (14.2)	15 (5.7)	22 (44.0)	20 (23.3)
Hypercholesterolaemia	22 (5.5)	2 (0.8)	6 (12.0)	14 (16.3)
Smoking	81 (20.2)	28 (10.6)	14 (28.0)	39 (45.3)
Positive family history	52 (13.0)	12 (4.5)	18 (36.0)	22 (25.6)
Known atherosclerotic disease	26 (6.5)	1 (0.4)	7 (14.0)	18 (20.9)
Number of risk factors (of six)	1 [0–2]	0 [0–1]	2 [1–3]	2 [1–3]

Data are median [IQR] or n (%). Percentages are column percentages. The seven cardiovascular conditions listed are not mutually exclusive, since a patient could have more than one, so their percentages do not sum to 100. The final row counts only the six conditions that contribute to the risk-factor component of the HEART score. Known atherosclerotic disease, although listed above with them, is not one of those six: the score counts it separately, treating it as equivalent to three or more risk factors. No *p*-values or effect sizes are reported; see text. HEART, History, Electrocardiogram, Age, Risk factors, Troponin; IQR, interquartile range.

**Table 2:** Distribution of the HEART score and its five components, overall and by risk category.

Characteristic	Total (n = 400)	Low Risk 0–3 (n = 264)	Moderate Risk 4–6 (n = 50)	High Risk 7–10 (n = 86)
***History***				
Slightly suspicious	279 (69.8)	252 (95.5)	27 (54.0)	0 (0.0)
Moderately suspicious	37 (9.2)	12 (4.5)	19 (38.0)	6 (7.0)
Highly suspicious	84 (21.0)	0 (0.0)	4 (8.0)	80 (93.0)
***Electrocardiogram***				
Normal	270 (67.5)	248 (93.9)	22 (44.0)	0 (0.0)
Non-specific repolarisation abnormality	41 (10.2)	16 (6.1)	19 (38.0)	6 (7.0)
Significant ST deviation	89 (22.2)	0 (0.0)	9 (18.0)	80 (93.0)
***Age category***				
<45 years	149 (37.2)	145 (54.9)	0 (0.0)	4 (4.7)
45–64 years	132 (33.0)	91 (34.5)	12 (24.0)	29 (33.7)
≥65 years	119 (29.8)	28 (10.6)	38 (76.0)	53 (61.6)
***Risk factors***				
No risk factor	182 (45.5)	180 (68.2)	2 (4.0)	0 (0.0)
1–2 risk factors	141 (35.2)	74 (28.0)	19 (38.0)	48 (55.8)
≥3 risk factors or atherosclerotic disease	77 (19.2)	10 (3.8)	29 (58.0)	38 (44.2)
***Troponin***				
≤ULN	306 (76.5)	262 (99.2)	44 (88.0)	0 (0.0)
1–3 × ULN	17 (4.2)	2 (0.8)	6 (12.0)	9 (10.5)
>3 × ULN	77 (19.2)	0 (0.0)	0 (0.0)	77 (89.5)
Total HEART score	2 [0–5]	1 [0–2]	5 [4–5]	9 [8–10]

Data are n (%) within each column, except the total score, which is median [IQR]. The risk category is computed from the five components shown, so their distribution across the categories reflects the construction of the score rather than an observed association, and no test or effect size is reported. The zero cells are observed rather than structural: every combination of a component level with a risk category is attainable in principle, since the total score depends on all five components. HEART, History, Electrocardiogram, Age, Risk factors, Troponin; ULN, upper reference limit; IQR, interquartile range.

**Table 3:** Cardiac troponin at presentation and at one hour, in relation to the laboratory reference interval.

Characteristic	Total (n = 400)	Low Risk 0–3 (n = 264)	Moderate Risk 4–6 (n = 50)	High Risk 7–10 (n = 86)
***Troponin at presentation (0 h)***				
At or below the upper reference limit	306 (76.5)	262 (99.2)	44 (88.0)	0 (0.0)
of which below the lower value of the reference interval	288 (72.0)	250 (94.7)	38 (76.0)	0 (0.0)
Above the upper reference limit	94 (23.5)	2 (0.8)	6 (12.0)	86 (100.0)
***Troponin at one hour (1 h)***				
At or below the upper reference limit	306 (76.5)	262 (99.2)	44 (88.0)	0 (0.0)
of which below the lower value of the reference interval	294 (73.5)	257 (97.3)	37 (74.0)	0 (0.0)
Above the upper reference limit	94 (23.5)	2 (0.8)	6 (12.0)	86 (100.0)
Elevated at either time point	96 (24.0)	3 (1.1)	7 (14.0)	86 (100.0)

Data are n (%) within each column. For the presentation measurement, the dichotomy at the ULN is definitional, since it determines the troponin component of the score; the division below that limit is not. The one-hour measurement does not enter the score. ULN, upper reference limit.

**Table 4:** Disposition and in-hospital course, by HEART risk category.

Characteristic	Total (n = 400)	Low Risk 0–3 (n = 264)	Moderate Risk 4–6 (n = 50)	High Risk 7–10 (n = 86)
Hospital admission	83 (20.8)	0 (0.0)	1 (2.0)	82 (95.3)
Length of stay, days, among admitted patients	4.0 [3.0–5.5] (n = 83)	—	2 (n = 1)	4.0 [3.0–5.8] (n = 82)
***Treatment during the index episode***				
No treatment	317 (79.2)	264 (100.0)	49 (98.0)	4 (4.7)
Conservative therapy	32 (8.0)	0 (0.0)	1 (2.0)	31 (36.0)
PCI	46 (11.5)	0 (0.0)	0 (0.0)	46 (53.5)
CABG	5 (1.2)	0 (0.0)	0 (0.0)	5 (5.8)
Revascularisation (PCI or CABG)	51 (12.8)	0 (0.0)	0 (0.0)	51 (59.3)
In-hospital death	5 (1.2)	0 (0.0)	0 (0.0)	5 (5.8)

Data are n (%) or median [IQR]. Length of stay is reported only among admitted patients, with the denominator given in each cell; the zeros in the remaining patients are structural rather than observed, and an em dash marks a category from which no patient was admitted. HEART, History, Electrocardiogram, Age, Risk factors, Troponin; IQR, interquartile range; PCI, percutaneous coronary intervention; CABG, coronary artery bypass grafting.

**Table 5:** Univariate associations with an elevated cardiac troponin concentration.

Characteristic	Elevated Among Those with It, n/N (%)	Elevated Among Those Without It, n/N (%)	RR or Cliff’s Delta (95% CI)	OR (95% CI)	*p*
Female sex	29/191 (15.2)	67/209 (32.1)	0.47 (0.32–0.70)	0.38 (0.23–0.62)	<0.001
Rural residence	35/151 (23.2)	61/249 (24.5)	0.95 (0.66–1.36)	0.93 (0.58–1.50)	0.765
Arterial hypertension	64/155 (41.3)	32/245 (13.1)	3.16 (2.18–4.59)	4.68 (2.87–7.64)	<0.001
Diabetes mellitus	29/58 (50.0)	67/342 (19.6)	2.55 (1.83–3.57)	4.10 (2.30–7.33)	<0.001
Obesity	22/57 (38.6)	74/343 (21.6)	1.79 (1.22–2.63)	2.28 (1.26–4.13)	0.005
Hypercholesterolaemia	14/22 (63.6)	82/378 (21.7)	2.93 (2.03–4.24)	6.32 (2.56–15.58)	<0.001
Smoking	41/81 (50.6)	55/319 (17.2)	2.94 (2.13–4.05)	4.92 (2.91–8.31)	<0.001
Positive family history	25/52 (48.1)	71/348 (20.4)	2.36 (1.66–3.35)	3.61 (1.98–6.60)	<0.001
Known atherosclerotic disease	19/26 (73.1)	77/374 (20.6)	3.55 (2.61–4.82)	10.47 (4.25–25.81)	<0.001
***Continuous characteristics***					
Age, years	68.0 [57.0–75.2]	46 [33–62]	+0.621 (+0.524 to +0.702; large)	—	<0.001
Systolic blood pressure, mmHg	150 [130–165]	140 [130–155]	+0.134 (−0.013 to +0.276; negligible)	—	0.047
Diastolic blood pressure, mmHg	81.5 [74.5–90.0]	80 [70–90]	+0.106 (−0.035 to +0.242; negligible)	—	0.115

The comparison includes all 400 patients, of whom 96 had an elevated cardiac troponin concentration. For categorical characteristics the cells give the number and proportion of patients with an elevated troponin concentration among those with and without the characteristic. Both the risk ratio and the odds ratio are shown: the outcome occurs in 24.0% of the cohort, so the odds ratio does not approximate the risk ratio and, taken alone, would overstate the association. For continuous characteristics the cells give the median [IQR] in each troponin group and the effect size is Cliff’s delta with its 95% confidence interval; the odds ratio column does not apply and an em dash is shown in its place. All expected cell counts exceeded five, so Pearson’s chi-squared test without continuity correction was used throughout for the categorical comparisons; continuous comparisons used the Mann–Whitney U test. RR, risk ratio; OR, odds ratio; CI, confidence interval; IQR, interquartile range.

**Table 6:** Exploratory multivariable logistic regression model for an elevated cardiac troponin concentration.

Covariate	Adjusted OR (95% CI)	*p*	Adjusted RR (95% CI)
Age, per 10 years	2.38 (1.88–3.09)	<0.001	1.43 (1.32–1.56)
Female sex	0.22 (0.11–0.41)	<0.001	0.47 (0.32–0.63)
Arterial hypertension	1.58 (0.86–2.94)	0.144	1.25 (0.92–1.78)
Diabetes mellitus	1.57 (0.77–3.21)	0.210	1.23 (0.87–1.74)
Smoking	4.10 (2.12–8.06)	<0.001	1.91 (1.40–2.66)
Known atherosclerotic disease	2.95 (1.02–9.27)	0.046	1.59 (0.88–2.63)

The model was fitted to all 400 patients, of whom 96 had an elevated concentration, giving 16.0 events per variable. Odds ratios are conditional estimates from the logistic model, with profile-likelihood confidence intervals and likelihood-ratio *p*-values, so that interval and test derive from the same likelihood. Risk ratios are marginal estimates obtained by standardising the fitted model over the cohort, with percentile confidence intervals from 5000 bootstrap replications. The two therefore estimate different quantities by different methods and need not agree on whether an interval excludes the null, as they do not for known atherosclerotic disease. Renal function was not recorded and could not be included in the model. OR, odds ratio; RR, risk ratio; CI, confidence interval.

## Data Availability

The original contributions presented in this study are included in the article. Further inquiries can be directed to the corresponding authors.

## Supplementary Materials

The following supporting information can be downloaded at https://www.mdpi.com/article/10.3390/jcm15187088/s1: Supplementary Methods S1: extended statistical methods—marginal standardisation of the fitted logistic model, the nonparametric bootstrap procedure, computation of E-values, the full model-diagnostic results, and the sensitivity of the counts to the troponin threshold; Table S1: standardised adjusted risk ratios and E-values for the exploratory multivariable model of cardiac troponin elevation; Table S2: number of patients with an elevated cardiac troponin concentration and number discharged from the emergency department with one at six thresholds; Table S3: the 13 patients discharged from the emergency department with a cardiac troponin concentration above the upper reference limit at one or both time points.
