# Supplementary material for: HEART Score Distribution and Association with In-Hospital Management and Outcomes in COVID-19 Survivors Presenting with Acute Chest Pain
Source: J Clin Med. 2026 Sep 13;15(18):7088. doi: 10.3390/jcm15187088 (PMC13607607; doi:10.3390/jcm15187088)
Supplement: Supplementary file 1 [file jcm-15-07088-s001.zip › jcm-4529263-supplementary.pdf]

Supplementary Materials

Clinical Utility of the HEART Score in COVID-19 Survivors with Acute Chest Pain: A Single-Center Retrospective Study

Supplementary Methods S1. Extended statistical methods

S1.1. Marginal standardisation of the fitted logistic model

The multivariable logistic regression model reported in Table 6 of the main text estimates conditional odds ratios. Because an elevated cardiac troponin concentration occurs in 24.0% of the cohort, the odds ratio does not approximate the risk ratio, and an approximate conversion would be unreliable. Adjusted risk ratios were therefore estimated directly by marginal standardisation. For each covariate, the fitted model was used to predict the risk of the outcome for every patient in the cohort under exposure and again under non-exposure, with all other covariates held at their observed values; the adjusted risk ratio was taken as the ratio of the two mean predicted risks. For age, the contrast is ten years. These standardised risk ratios are marginal estimates and therefore address a different quantity from the conditional odds ratios reported in the main text.

S1.2. Bootstrap confidence intervals

Confidence intervals for the standardised risk ratios were obtained by nonparametric bootstrap over patients, refitting the model in each replication, using the percentile method with 5000 replications and a fixed random seed (20260807). No replication failed to converge, and all 5000 were used; complete separation arose in 3 of the 5000 replications, a proportion too small to affect the percentile intervals materially. Because the odds ratios are conditional estimates with profile-likelihood intervals whereas the standardised risk ratios are marginal estimates with bootstrap intervals, the two need not agree on whether a given interval excludes the null; where they differ, both are reported.

S1.3. E-values

The E-value gives the minimum strength of association, on the risk-ratio scale, that an unmeasured confounder would need to have with both the covariate and the outcome in order to move the estimate to the null, conditional on the assumptions of the E-value framework. Because the E-value is defined on the risk-ratio scale, it was computed from the standardised risk ratios described in S1.1 rather than from the odds ratios. E-values are reported for each standardised risk ratio and for the confidence limit closest to the null; a value of 1.00 for the latter denotes an interval that already includes the null, for which no confounding is required.

These quantities measure sensitivity to confounding under the assumptions of that framework. They do not establish that an association is free of confounding and, in particular, they cannot compensate for the absence of renal function from the model. The E-value is defined for a causal contrast, whereas the model reported here is exploratory and estimates adjusted associations; it is therefore presented only as a supplementary sensitivity analysis, showing how fragile a causal reading of these estimates would be, and not as a basis for causal inference. For age and sex, which are not modifiable exposures, a causal contrast is not well defined, and the corresponding E-values should be read with that reservation.

S1.4. Model diagnostics

Model assumptions were examined and none was violated. Linearity of age on the logit scale was assessed by the Box–Tidwell procedure ($p = 0.796$). Multicollinearity was assessed by variance inflation factors, all of which were ≤ 1.42. Influential observations were assessed by Cook's distance, the maximum value being 0.059. Calibration was assessed by the Hosmer–Lemeshow test, which did not detect a departure from calibration ($\chi^2 = 9.83$, 8 degrees of freedom, $p = 0.277$). The Hosmer–Lemeshow test has limited power and relies on arbitrary decile grouping, so a non-significant result indicates only that no departure from calibration was detected, not that calibration is good.

S1.5. Sensitivity of the counts to the troponin threshold

The number of patients classified as having an elevated cardiac troponin concentration, and the number discharged from the emergency department with such a concentration, both depend on the threshold applied. The threshold used throughout the main text is the upper reference limit of the local laboratory (29 ng/L). Because the lower value of that laboratory reference interval is not consistent with the observed distribution, both counts were recomputed at six thresholds spanning approximately seven-tenths to approximately one and eight-tenths of the upper reference limit, which is itself among them (Table S2). The thresholds were chosen after inspecting the distribution; the analysis is therefore post hoc and exploratory, the counts are reported descriptively, and no test of trend was performed. Proportions are given with exact (Clopper–Pearson) 95% confidence intervals.

Table S1. Standardised adjusted risk ratios and E-values for unmeasured confounding, exploratory multivariable model of an elevated cardiac troponin concentration.

Covariate	Adjusted OR (95% CI)	Adjusted RR (95% CI)	E-value	E-value, CI limit
Age, per 10 years	2.38 (1.88–3.09)	1.43 (1.32–1.56)	2.22	1.98
Female sex	0.22 (0.11–0.41)	0.47 (0.32–0.63)	3.70	2.53
Arterial hypertension	1.58 (0.86–2.94)	1.25 (0.92–1.78)	1.80	1.00
Diabetes mellitus	1.57 (0.77–3.21)	1.23 (0.87–1.74)	1.77	1.00
Smoking	4.10 (2.12–8.06)	1.91 (1.40–2.66)	3.23	2.15
Known atherosclerotic disease	2.95 (1.02–9.27)	1.59 (0.88–2.63)	2.56	1.00

The model was fitted to all 400 patients, of whom 96 had an elevated cardiac troponin concentration at presentation or at one hour, giving 16.0 events per variable. Odds ratios are conditional estimates from the logistic model, with profile-likelihood confidence intervals. Risk ratios are marginal estimates obtained by standardising the fitted model over the cohort, with percentile confidence intervals from 5000 bootstrap replications; the two therefore estimate different quantities by different methods and need not agree on whether an interval excludes the null, as they do not for known atherosclerotic disease. E-values are computed from the standardised risk ratio and give the minimum strength of association that an unmeasured confounder would need to have with both the covariate and the outcome in order to move the estimate to the null; the final column gives the corresponding value for the confidence limit closest to the null. An E-value of 1.00 for that limit indicates an interval that already includes the null, for which no confounding is required. Renal function was not recorded and could not be included in the model. This analysis is exploratory and does not support causal inference. OR, odds ratio; RR, risk ratio; CI, confidence interval.

Table S2. Number of patients with an elevated cardiac troponin concentration, and number discharged from the emergency department with one, at six thresholds.

Threshold, ng/L	Multiple of the ULN	Elevated at either time point, n (%)	Discharged with an elevated concentration, n/N (%; 95% CI)
20	0.69	100 (25.0)	17/317 (5.36; 3.15–8.45)
26	0.90	96 (24.0)	13/317 (4.10; 2.20–6.91)
29 (ULN)	1.00	96 (24.0)	13/317 (4.10; 2.20–6.91)
34	1.17	91 (22.8)	8/317 (2.52; 1.10–4.91)
45	1.55	90 (22.5)	7/317 (2.21; 0.89–4.50)
52	1.79	90 (22.5)	7/317 (2.21; 0.89–4.50)

All 400 patients contribute to the third column and all 317 patients discharged from the emergency department to the fourth. A patient is counted as elevated if the concentration exceeded the threshold at presentation, at one hour, or at both. The threshold of 29 ng/L is the upper reference limit (ULN) of the local laboratory and is the one applied throughout the main text; the remaining five were chosen after inspecting the observed distribution, so this analysis is post hoc and exploratory. Percentages in the fourth column are accompanied by exact (Clopper–Pearson) 95% confidence intervals. The counts are reported descriptively and no test of trend was performed. Across the whole range of thresholds examined, between 7 and 17 of the 317 discharged patients had an elevated concentration, so the observation that a small number of

patients were discharged with an elevated troponin concentration does not depend on the particular threshold adopted. ULN, upper reference limit; CI, confidence interval.

Table S3. The 13 patients discharged from the emergency department with a cardiac troponin concentration above the upper reference limit at one or both time points.

Patient	Age category	Sex	HEART score	Risk category	H	E	A	R	Tn	Troponin 0 h	Troponin 1 h
1	<45 years	M	1	Low risk (0–3)	0	0	0	0	1	1–2 × ULN	≤ ULN
2	45–64 years	F	2	Low risk (0–3)	0	0	1	1	0	≤ ULN	1–2 × ULN
3	45–64 years	M	3	Low risk (0–3)	0	0	1	1	1	2–5 × ULN	2–5 × ULN
4	45–64 years	M	4	Moderate risk (4–6)	1	0	1	1	1	1–2 × ULN	≤ ULN
5	≥65 years	M	4	Moderate risk (4–6)	0	0	2	2	0	≤ ULN	1–2 × ULN
6	45–64 years	M	5	Moderate risk (4–6)	1	0	1	2	1	1–2 × ULN	2–5 × ULN
7	≥65 years	M	5	Moderate risk (4–6)	1	0	2	1	1	1–2 × ULN	1–2 × ULN
8	45–64 years	F	5	Moderate risk (4–6)	1	0	1	2	1	1–2 × ULN	2–5 × ULN
9	≥65 years	F	5	Moderate risk (4–6)	0	1	2	1	1	1–2 × ULN	2–5 × ULN
10	≥65 years	M	7	High risk (7–10)	1	1	2	2	1	1–2 × ULN	2–5 × ULN
11	≥65 years	M	7	High risk (7–10)	1	1	2	2	1	1–2 × ULN	1–2 × ULN
12	≥65 years	M	8	High risk (7–10)	1	1	2	2	2	> 5 × ULN	> 5 × ULN
13	≥65 years	M	8	High risk (7–10)	1	1	2	2	2	> 5 × ULN	> 5 × ULN

These 13 patients are the 4.10% (95% CI 2.20–6.91) of the 317 patients discharged from the emergency department who had a cardiac troponin concentration above the upper reference limit at presentation, at one hour, or at both; they are the patients marked by triangles in Figure 3 of the main text. Columns H, E, A, R and Tn give the points scored on each of the five HEART score components (History, Electrocardiogram, Age, Risk factors, Troponin), which sum to the total HEART score. The troponin columns give the measured concentration expressed as a multiple of the upper reference limit; note that these categories are finer than the three-level troponin component of the score, and that the one-hour measurement does not enter the score. Post-discharge outcomes were not available, so no judgement about the safety or appropriateness of these discharge decisions can be made from these data; the listing is presented to document the composition of this subgroup. ULN, upper reference limit.
